# Classification of stable solutions for non-homogeneous higher-order elliptic PDEs

**DOI:** 10.1186/s13660-017-1352-9

**Published:** 2017-04-18

**Authors:** Abdellaziz Harrabi, Belgacem Rahal, Mohamed Karim Hamdani

**Affiliations:** 1 Institut Supérieur des Mathématiques Appliquées et de l’Informatique de Kairouan, Université de Kairouan, Kairouan, Tunisia; 2 Faculté des Sciences, Département de Mathématiques, Université de Sfax, B.P. 1171, 3000, Sfax, Tunisia

**Keywords:** 35J55, 35J65, 35B33, 35B65, higher-order equation, Liouville theorems, finite Morse index, Pohozaev identity

## Abstract

Under some assumptions on the nonlinearity *f*, we will study the nonexistence of nontrivial stable solutions or solutions which are stable outside a compact set of $\mathbb {R}^{n}$ for the following semilinear higher-order problem:

$$\begin{aligned} (-\Delta)^{k} u= f(u) \quad \mbox{in }\mathbb {R}^{n}, \end{aligned}$$

with $k=1,2,3,4$. The main methods used are the integral estimates and the Pohozaev identity. Many classes of nonlinearity will be considered; even the sign-changing nonlinearity, which has an adequate subcritical growth at zero as for example $f(u)= -m u +\lambda|u|^{\theta-1}u-\mu |u|^{p-1}u$, where $m\geq0$, $\lambda>0$, $\mu>0$, $p, \theta>1$. More precisely, we shall revise the nonexistence theorem of Berestycki and Lions (Arch. Ration. Mech. Anal. 82:313-345, 1983) in the class of smooth finite Morse index solutions as the well known work of Bahri and Lions (Commun. Pure Appl. Math. 45:1205-1215, 1992). Also, the case when $f(u)u$ is a nonnegative function will be studied under a large subcritical growth assumption at zero, for example $f(u)=|u|^{\theta-1}u(1 + |u|^{q})$ or $f(u)= |u|^{\theta-1}u e^{|u|^{q}}$, $\theta>1$ and $q>0$. Extensions to solutions which are merely stable are discussed in the case of supercritical growth with $k=1$.

## Introduction

This paper is devoted to the study of solutions, possibly unbounded and sign-changing, of the semilinear partial differential equation,

1.1 $$\begin{aligned} (-\Delta)^{k} u= f(u) \quad \mbox{in } \mathbb {R}^{n} , \end{aligned}$$

where $k=1,2,3,4$, $n\geq1$ and $f\in C^{1}(\mathbb {R})$. Under some assumptions on the nonlinearity *f*, we will show that this problem does not possess a nontrivial solution with finite Morse index.

In the last decades, problems related to the nonexistence of finite Morse index solutions for second-, fourth- and sixth-order Lane-Emden equation on unbounded domains of $\mathbb {R}^{n}$ have received a lot of attention (see [2–12]).

We now list some known results. We start with the second-order Lane-Emden equation

1.2 $$\begin{aligned} -\Delta u=|u|^{p-1}u, \quad \mbox{in } \mathbb {R}^{n}, p>1, \end{aligned}$$

Farina [6] proved that nontrivial finite Morse index solutions of (1.2) exist if and only if $p\geq p_{c}(n)$ and $n\geq 11$, or $p=\frac{n+2}{n-2}$ and $n\geq3$, where $p_{c}(n) $ is the so-called Joseph-Lundgren exponent. Also, in [13] several Liouville-type theorems are presented for stable solutions, where $f>0$ is a general convex, nondecreasing function. Extensions to solutions which are merely stable outside a compact set are discussed.

For the fourth-order Lane-Emden problem

1.3 $$\begin{aligned} \Delta^{2} u=|u|^{p-1}u, \quad \mbox{in } \mathbb {R}^{n}, p>1, \end{aligned}$$

the subcritical case has been studied by Ramos and Rodriguez for finite Morse index sign-changing solutions (see [14]). The supercritical case is more complicated and there are several new approaches dealing with (1.3). The first approach is to use the test function $v=-\Delta u$. To this end, one has to use Souplet’s inequality [15], this will give an exponent $\frac{n}{n-8}+ \epsilon_{n}$ for some $\epsilon_{n}>0$; see [16]. These results were improved in [12] by adapting Farina’s approach with the restriction on the power $q<\frac{2}{3}$. The second approach was obtained by Cowan and Ghoussoub [3], Dupaigne *et al.* [17] and further exploited by Hajlaoui, Ye and one of the authors [7]. This approach improves the first upper bound $\frac {n}{n-8}+ \epsilon_{n}$, but it again fails to catch the fourth-order Joseph-Lundgren exponent computed by Gazzola and Grunau [18]. It should be remarked that by combining these two approaches one can show that stable positive solutions to (1.3) do not exist when $n\leq12$ and $p>1$; see [7]. Finally in [5], Dávila *et al.* employed a monotonicity formula-based approach and gave a complete classification of stable and finite Morse index (positive or sign-changing) solutions to (1.3). A remarkable outcome of this third approach is that it gives the optimal exponent. The main tool of [5] is a monotonicity formula, used to perform a blow-down analysis and reduce the nonexistence of nontrivial entire solutions for the problem (1.3), to that of nontrivial homogeneous solutions.

Thanks to the Liouville-type theorem with finite Morse index in [8], the authors proved the nonexistence result of sign-changing solutions for the sixth-order problem

1.4 $$\begin{aligned} -\Delta^{3} u=|u|^{p-1}u, \quad \mbox{in } \mathbb {R}^{n}, p>1. \end{aligned}$$

Let us give a brief sketch of their method. They proved various classification theorems and Liouville-type results for $C^{6}$-solutions belonging to one of the following classes: stable solutions, solutions which are stable outside a compact set of $\mathbb {R}^{n}$. These results apply to the subcritical case using the Pohozaev identity. In the supercritical case, motivated by the monotonicity formula established in [19], they reduced the nonexistence of nontrivial entire solutions for the problem (1.4), to that of nontrivial homogeneous solutions. Through this approach, they gave a complete classification of stable solutions and those finite Morse indices, whether positive or sign-changing. Also, this analysis reveals the existence of a new critical exponent called the sixth-order Joseph-Lundgren exponent, also they gave the explicit value of this exponent.

In this work, we are concerned with Liouville-type theorems for the nonlinear elliptic equation (1.1) for $k=1,2,3,4$. We prove Liouville-type theorems for solutions (whether positive or sign-changing) belonging to one of the following classes: stable solutions and solutions which are stable outside a compact set. Our proof is based on a combination of the integral estimates and the Pohozaev-type identity.

The paper is organized as follows. In Section 2 we state our main results, which are then proved in Section 4. Section 3 contains some important auxiliary tools, which are used in the proofs of the main theorems.

## Statement of the main results

In order to state our results, we present first some assumptions on the nonlinearity *f*:

$H_{1}$: There exists a constant $\theta>1 $ such that

$$f'(s) s^{2} -\theta f(s) s \geq0 ,\quad\forall s\in \mathbb {R}. $$

$H_{2}$: There exist constants $s_{0}>0$, $\theta>1$ and $C_{0}>0$ such that

$$C_{0} |s|^{\theta+1 } \leq f(s) s, \quad \forall |s|\leq s_{0}. $$

$H_{3}$: There exists a constant $0< \alpha_{0}< 1$ such that

$$\begin{aligned} \frac{2n}{n-2k} F(s) -(1+\alpha_{0}) f(s) s \geq0,\quad \forall s \in \mathbb {R}, \end{aligned}$$

where $F(s)= {\int_{0}^{s}} f(t)\,dt$.

### Remark 2.1

(1) $H_{1}$ implies $H'_{1}$: There exist constants $s_{0}>0$, $\theta>1$ and $C_{0}>0$ such that

$$C_{0} |s|^{\theta+1 } \leq f(s) s, \quad \forall |s|\geq s_{0}. $$

Indeed, by $H_{1}$, we have $\frac{f}{|s|^{\theta}}$ is nondecreasing function for all $|s|\geq s_{0}$. This implies that

$$C_{0} |s|^{\theta+1 } \leq f(s) s,\quad \forall |s|\geq s_{0}. $$

(2) $H_{1}$ implies the Ambrosetti-Rabinowitz condition (A-R): there exist constants $\widetilde{\theta}>2$ and $s_{0}>0$ such that

$$f(s) s \geq\widetilde{\theta} F(s) > 0,\quad \mbox{for } |s|>s_{0}. $$

### Examples

We easily verify that the following functions satisfy $H_{1}$ and $H_{2}$.

1. $f(s)= C_{0} ( 1+ |s|^{q} )|s|^{\theta-1}s$, $\theta >1$, $q>0$ and $C_{0}>0$.
2. $f(s)= |s|^{\theta-1}s e^{|s|^{q}} $, $\theta>1$ and $q>1$.
3. $f(s)=\sum_{i=1}^{i=i_{0}} c_{i} |s|^{\theta_{i}-1}s$, with $\theta_{i}>1$ $\forall i=1,2,\ldots, i_{0}$ and $c_{i}>0$ $\forall i=1,2,\ldots, i_{0}$. In this example we choose $\theta= \min_{1 \leq i \leq i_{0}} (\theta_{i})$.

The examples (1) and (2) show that *f* can have an exponential growth at infinity. Therefore, clearly an adequate behavior of *f* at zero is needed to obtain the Liouville theorem. The unique and important nonexistence result for stable solutions of the non-homogeneous second-order equation (1.1) has been recently obtained in [13]. It is shown there, among other things, that (1.1) does not admit nontrivial stable or stable outside a compact set solution provided that *f* is regular, positive, nondecreasing and convex function in $(0, +\infty)$. More precisely, under a mere nonnegativity assumption on the nonlinearity, the authors begin this work by stating that up to space dimension $n=4$, bounded stable solutions of (1.1) are trivial. For the next series of results, they restricted themselves to the following class of nonlinearities:

2.1 $$\begin{aligned} f \in C^{0}(\mathbb {R}_{+}) \cap C^{2}\bigl(\mathbb {R}^{*}_{+} \bigr),\quad f > 0 \mbox{ is nondecreasing and convex in } \mathbb {R}^{*}_{+}. \end{aligned}$$

In order to relate the nonlinearity *f* and the below exponents (2.3) and (2.4), they introduced a quantity *q* defined for $u \in \mathbb {R}^{*}_{+} $ by $q(u)=\frac{f^{\prime 2}}{ff''}(u)$, whenever $ff''(u)\neq0$ and $q(u)=+\infty$ otherwise. They assumed that $q(u)$ converges as $u \rightarrow0^{+}$ and denote its limit by

2.2 $$\begin{aligned} q_{0}=\lim_{u\rightarrow0^{+}}q(u) \in\overline{\mathbb {R}}. \end{aligned}$$

Define now $p_{0} \in\overline{\mathbb {R}}$ the conjugate exponent of $q_{0}$, by $\frac{1}{p_{0}} + \frac{1}{q_{0}}=1$. The exponent $p_{0}$ must be understood as a measure of the ’flatness’ of *f* at 0. However, we establish their following theorem.

### Theorem A

[13] *Assume that* *f* *satisfies* (2.1) *and* (2.2). *Assume that* $u \in C^{2}(\mathbb {R}^{n})$ *is stable solution of* (1.1) *with* $k=1$. *Then* $u \equiv0$ *if any one of the following conditions holds*:

1. $1\leq n\leq9$ *and* $1<\underline{p_{\infty}}$,
2. $n=10$, $p_{0} <+\infty$ *and* $1<\underline{p_{\infty}}$,
3. $n\geq 11$, $p_{0}< p_{c}(n)$ *and* $1<\underline{p_{\infty}} <p_{c}(n)$,

*where* $\underline{p_{\infty}} \in\overline{\mathbb {R}}$ *be defined by* $\overline{q_{\infty}}= {\limsup_{u \rightarrow+\infty }} q(u)$, $\frac{1}{\underline{p_{\infty}}}+ \frac{1}{\overline {q_{\infty}}}=1$.

A typical example of nonlinearity function *f* satisfying the above conditions (2.1) and (2.2) is $f(u)=|u|^{\theta -1}u+|u|^{p-1}u$, where $p\geq\theta$. A simple calculation, we get $p_{0}=\theta$ and $\underline{p_{\infty}}=p$. We use this nonlinearity function to establish some new Liouville-type theorems. Our method is different from (and complementary to) the one used in [13]. It exploits the attractive character of the difference between $f'(u) u^{2}- \theta f(u) u\geq0$, if $p\geq\theta$, that is, *f* satisfies $H_{1}$ and $H_{2}$. It will be shown in Theorem 2.1 that problem (1.1) does not possess nontrivial stable solutions if and only if $1<\theta<p_{c}(n) $, $\forall p\geq\theta $. Also, we may consider nonlinearities with exponential growth at infinity, *i.e.* $\underline{p_{\infty}}=\infty$ satisfying $H_{1}$ and $H_{2}$, as for example $f(u)= |u|^{\theta-1}u e^{|u|^{q}} $, $\theta>1$ and $q>0$; therefore, in view again of Theorem 2.1, one has $u\equiv0$. Furthermore, the present paper is motivated by the interesting work [1], we shall revise the nonexistence theorem of Berestycki and Lions [1] if one substitutes their assumption, which is

$$\int_{\mathbb {R}^{n}}|\nabla u|^{2}+ \int_{\mathbb {R}^{n}}f(u)u< +\infty, $$

by assuming that *u* is stable or stable outside a compact set. Therefore sign-changing nonlinearities will also be considered and we do not require that $f'(0)=0$ as the instructive example given by Berestycki and Lions [1] is $f(u)= -m u +\lambda |u|^{\theta-1}u-\mu|u|^{p-1}u$, where $\lambda,\mu$ are positive constants, $m\geq0$ and $1<\theta,p$. Observe that the above nonlinearity satisfies ($H_{1}$), thus we shall prove that equation (1.1) does not possess a nontrivial stable solution provided $1< p \leq\frac{n+2k}{n-2k}$ and $p<\theta$, also if *u* is bounded solution to (1.1) and $m>0$, then $u\equiv0$, for any $\theta\geq p$. If $p \leq\frac{n+2k}{n-2k}\leq\theta$ and $m>0$, it follows from the Pohozaev identity that there cannot exist a nontrivial solution of (1.1) which is stable outside a compact set. This result is similar to [1] for $k=1$. To conclude, this work completes the study of Dupaigne and Farina [13] since here we do not assume that *f* is positive and convex function. Therefore, to be more concrete in our analysis of nonexistence, we will distinguish between stable and stable outside a compact set. We provide some elliptic decay estimates that we use frequently later in the proofs. Deriving the right decay estimates for solutions of (1.1) plays a fundamental role in most our proofs. On the other hand, we shall also consider the question of the nonexistence of stable solutions (positive or sign-changing) in the supercritical case of a second-order equation.

In order to state our results we need to recall the following.

### Definition 2.1

A solution *u* of (1.1) belonging to $C^{2k}(\mathbb {R}^{n})$

- is said to be stable if
  $$Q_{u}(\psi):= \int_{\mathbb {R}^{n}} \bigl\vert D^{k} \psi \bigr\vert ^{2} \,dx - \int_{\mathbb {R}^{n}} f'(u)\psi^{2} \,dx \geq0 , \quad \forall \psi\in C_{c}^{k}\bigl( \mathbb {R}^{n}\bigr), $$
  where
  $$D^{k}= \textstyle\begin{cases} \Delta^{\frac{k}{2}} &\text{for $k=2,4$,}\\ \nabla\Delta^{\frac{k-1}{2}} &\text{for $k=1,3$,} \end{cases} $$
- is stable outside a compact set $\mathcal{K}\subset \mathbb {R}^{n}$, if $Q_{u} (\psi)\geq0$ for any $\psi\in C^{k}_{c}(\mathbb {R}^{n} \backslash\mathcal {K})$.
  More generally, the Morse index of a solution is defined as the maximal dimension of all subspaces *E* of $C^{k}_{c}(\mathbb {R}^{n})$ such that $Q_{u} (\psi)< 0$ in $E\backslash\{0\}$. Clearly, a solution is stable if and only if its Morse index is equal to zero.

### Remark 2.2

It is well known that any finite Morse index solution *u* is stable outside a compact set $\mathcal{K}\subset \mathbb {R}^{n}$. Indeed, there exist $K\geq1$ and $X_{K}:= \operatorname{Span}\{\phi_{1},\ldots, \phi_{K}\} \subset C^{k}_{c}(\mathbb {R}^{n})$ such that $Q_{u} (\phi)<0$ for any $\phi\in X_{K}\backslash\{0\}$. Hence, $Q_{u} (\psi)\geq0$ for every $\psi\in C^{k}_{c}(\mathbb {R}^{n}\backslash \mathcal{K}) $, where $\mathcal{K}:= \bigcup_{j=1}^{K} \operatorname{supp}(\phi_{j})$.

To state the following result we need to introduce some notation. Let two critical exponents play an important role, namely the classical Sobolev exponent

2.3 $$\begin{aligned} p_{s}(n,k)= \textstyle\begin{cases} +\infty &\text{if $n\leq2k$},\\ \frac{n+2k}{n-2k} &\text{if $n> 2k$}, \end{cases}\displaystyle \end{aligned}$$

and the Joseph-Lundgren exponent

2.4 $$\begin{aligned} p_{c}(n)= \textstyle\begin{cases} +\infty &\text{if $n\leq10$},\\ \frac{(n-2)^{2}-4n+ 8\sqrt{n-1}}{(n-2)(n-10)} &\text{if $n\geq11$}. \end{cases}\displaystyle \end{aligned}$$

Note that the exponent $p_{c}(n)$ is larger than the classical critical Sobolev exponent $p_{s}(n,1)$, $n\geq11$.

Now we can state our main nonexistence results.

### Theorem 2.1

*Let* $u\in C^{2k}(\mathbb {R}^{n})$ *be a stable solution of* (1.1). *Assume that* *f* *satisfies* $H_{1}$ *and* $H_{2}$. *If* $1<\theta\leq p_{s}(n,k)$, *then* $u\equiv0$.

### Theorem 2.2

*Let* $u\in C^{2k}(\mathbb {R}^{n})$ *be a solution of* (1.1) *which is stable outside a compact set*. *Assume that* *f* *satisfies* $H_{1}$, $H_{2}$ *and* $H_{3}$. *If* $1<\theta< p_{s}(n,k)$, *then* $u\equiv0$.

The next result concerns the complete classification of entire stable solutions of the second-order equation (1.1) in the supercritical case.

### Theorem 2.3

*Let* $u\in C^{2}(\mathbb {R}^{n})$ *be a stable solution of* (1.1) *with* $k=1$. *Assume that* *f* *satisfies* $H_{1}$ *and* $H_{2}$. *If* $\frac {n+2}{n-2}<\theta< p_{c}(n)$, *then* $u\equiv0$.

### Berestycki and Lions Liouville-type theorem

Now, we fix in this subsection

2.5 $$\begin{aligned} f(u)=-m u + \lambda|u|^{\theta-1}u- \mu|u|^{p-1}u, \end{aligned}$$

where $\lambda,\mu$ are positive constants, $m\geq0$ and $1<\theta,p$. We will show that $u=0$ is the unique solution of equation (1.1) under some assumptions on the parameter *m*, *θ* and *p*. Also, we observe that *f* is neither convex nor positive function in $\mathbb {R}^{n}$. Then we have the following.

#### Theorem 2.4

*Let* $u\in C^{2k}(\mathbb {R}^{n})$ *be a stable solution of* (1.1) *with* *f* *satisfies* (2.5).

1. *If* *u* *is bounded and* $m>0$, *then* $u\equiv0$, *for any* $\theta\geq p>1$.
2. *If* $1< p < \theta$ *and* $1< p \leq p_{s}(n,k)$, *then* $u\equiv0$.

#### Remark 2.3

Clearly, if *u* is unbounded stable solution to (1.1) with $f(u)=-m u + \lambda|u|^{\theta-1}u- \mu|u|^{p-1}u$ and $m>0$, then $u\equiv0$, for any $\theta\geq p>1$ and $n<2k$.

Also, we will show, with very few restrictions, that there exists a necessary and sufficient condition for the nonexistence solutions which are stable outside a compact set of problem like (1.1).

#### Theorem 2.5

*Let* $u\in C^{2k}(\mathbb {R}^{n})$ *be a solution of* (1.1) *which is stable outside a compact set with* *f* *satisfies* (2.5).

1. *If* $m>0$ *and* $1< p \leq\frac{n+2k}{n-2k} \leq\theta$, *then* $u\equiv0$.
2. *If* $m=0$, $1< p \leq\frac{n+2k}{n-2k}\leq\theta$ *and* $(p, \theta)\neq(\frac{n+2k}{n-2k}, \frac{n+2k}{n-2k})$, *then* $u\equiv0$.

## Auxiliary results

In this section we prove the following lemmas and propositions, which will have a crucial role in the proof of Theorems 2.1, 2.2, 2.3, 2.4 and 2.5. Denote $B_{R}= \{x\in \mathbb {R}^{n}: |x|< R\}$. The letter *C* will be used throughout to denote a generic positive constant, which may vary from line to line and only depends on arguments inside the parentheses or arguments which are otherwise clear from the context.

First, define a cut-off function $\varphi_{ R} \in C^{4}_{c}(\mathbb {R}^{n})$ such that $\varphi_{ R}\equiv1$ in $B_{R}$, $\varphi_{ R}\equiv0 $ in $\mathbb {R}^{n} \backslash\{ B_{2R}\}$, $0\leq \varphi_{R}\leq1$ in $\mathbb {R}^{n}$ and $|\nabla^{\tau} \varphi_{ R}| \leq C R^{-\tau}$ for $\tau\leq4$ in $A_{R}=\{x \in \mathbb {R}^{n}, R \leq |x| \leq2 R \}$.

### Lemma 3.1

*For any* $v\in C^{8}(\mathbb {R}^{n})$, $m> 4$ *and* $\epsilon>0$ *arbitrary small number*, *there exists a constant* $C_{\epsilon,m}>0$ *such that*

1. $R^{-4} \int_{B_{2R}} |\Delta v|^{2}\varphi_{R}^{2m-4} \,dx\leq\epsilon^{2} \int_{B_{2R}}(\Delta^{2} v)^{2}\varphi_{R}^{2m} \,dx+\epsilon^{2}R^{-2} {\int_{B_{2R}}}|\nabla(\Delta v)|^{2}\varphi_{R}^{2m-2} \,dx+ C_{\epsilon,m} R^{-8} \int _{B_{2R}} v^{2}\varphi_{R}^{2m-8} \,dx$,
2. $R^{-2} {\int_{B_{2R}}}|\nabla(\Delta v)|^{2}\varphi_{R}^{2m-2} \,dx\leq \epsilon {\int_{B_{2R}}}(\Delta^{2} v)^{2}\varphi _{R}^{2m} \,dx+C_{\epsilon,m} R^{-8} {\int_{B_{2R}}}v^{2}\varphi _{R}^{2m-8} \,dx$,
3. $R^{-6} {\int_{B_{2R}}}|\nabla v|^{2}\varphi_{R}^{2m-6} \,dx\leq\epsilon^{3} {\int_{B_{2R}}}(\Delta^{2} v)^{2}\varphi _{R}^{2m} \,dx+C_{\epsilon,m} R^{-8} {\int_{B_{2R}}} v^{2}\varphi _{R}^{2m-8} \,dx$,
4. $R^{-4} {\int_{B_{2R}}}|\nabla^{2} v|^{2}\varphi_{R}^{2m-4} \,dx\leq\epsilon^{3} {\int_{B_{2R}}}(\Delta^{2} v)^{2}\varphi _{R}^{2m} \,dx+C_{\epsilon,m} R^{-8} {\int_{B_{2R}}} v^{2}\varphi _{R}^{2m-8} \,dx$,
5. $R^{-2}\int_{B_{2R}}|\nabla^{3} v|^{2}\varphi_{R}^{2m-2} \,dx \leq\epsilon ^{3} {\int_{B_{2R}}}(\Delta^{2} v)^{2}\varphi_{R}^{2m} \,dx+C_{\epsilon,m} R^{-8} {\int_{B_{2R}}} v^{2}\varphi _{R}^{2m-8} \,dx$.

### Proof

Fix $m> 4$. Let $v\in C^{8}(\mathbb {R}^{n})$ and $\varphi_{R} \in C^{4}_{c}(\mathbb {R}^{n})$ defined as above.

*Proof of* 1. Integrating by parts, we get

3.1 $$\begin{aligned}& R^{-4} \int_{B_{2R}}(\Delta v )^{2} \varphi_{R}^{2m-4} \,dx \\ & \quad= R^{-4} \int _{B_{2R}} v \bigl( \Delta^{2} v \varphi_{R}^{2m-4} + \Delta v\Delta \bigl(\varphi_{R}^{2m-4} \bigr) + 2\nabla(\Delta v)\nabla\bigl(\varphi_{R}^{2m-4}\bigr) \bigr) \,dx. \end{aligned}$$

An application of Young’s inequality yields

$$\begin{aligned}& R^{-4} \int_{B_{2R}} v \bigl( \Delta^{2} v \varphi_{R}^{2m-4} + \Delta v\Delta\bigl(\varphi_{R}^{2m-4} \bigr) + 2\nabla(\Delta v)\nabla\bigl(\varphi _{R}^{2m-4}\bigr) \bigr) \,dx\\ & \quad\leq \epsilon^{2} \int_{B_{2R}}\bigl(\Delta^{2} v\bigr)^{2} \varphi_{R}^{2m} \,dx+\epsilon^{2}R^{-2} \int_{B_{2R}} \bigl|\nabla(\Delta v)\bigr|^{2} \varphi_{R}^{2m-2} \,dx \\ & \qquad{}+ \frac{ R^{-4}}{2} \int _{B_{2R}}(\Delta v)^{2}\varphi_{R}^{2m-4} \,dx + C_{\epsilon,m} R^{-8} \int _{B_{2R}}v^{2}\varphi_{R}^{2m-8} \,dx. \end{aligned}$$

Inserting the latter inequality into (3.1), we obtain

3.2 $$\begin{aligned} R^{-4} \int_{B_{2R}}(\Delta v )^{2} \varphi_{R}^{2m-4} \,dx \leq &\epsilon^{2} \int_{B_{2R}}\bigl(\Delta^{2} v\bigr)^{2} \varphi_{R}^{2m} \,dx+\epsilon^{2}R^{-2} \int _{B_{2R}} \bigl|\nabla(\Delta v)\bigr|^{2} \varphi_{R}^{2m-2} \,dx \\ &{}+ C_{\epsilon,m} R^{-8} \int_{B_{2R}}v^{2}\varphi_{R}^{2m-8} \,dx. \end{aligned}$$

*Proof of* 2. Integrating by parts and using again Young’s inequality, we obtain

$$\begin{aligned}& R^{-2} \int_{B_{2R}} \bigl\vert \nabla(\Delta v) \bigr\vert ^{2}\varphi_{R}^{2m-2} \,dx \\ & \quad= -R^{-2} \int _{B_{2R}}\Delta v\Delta^{2} v\varphi_{R}^{2m-2} \,dx-R^{-2} \int _{B_{2R}}\Delta v\nabla(\Delta v)\nabla\bigl( \varphi_{R}^{2m-2}\bigr) \,dx \\ & \quad\leq \epsilon \int_{B_{2R}}\bigl(\Delta^{2} v\bigr)^{2} \varphi_{R}^{2m} \,dx+\frac {2R^{-4}}{\epsilon} \int_{B_{2R}}(\Delta v)^{2}\varphi_{R}^{2m-4} \,dx+ C\epsilon R^{-2} \int_{B_{2R}} \bigl\vert \nabla(\Delta v) \bigr\vert ^{2}\varphi_{R}^{2m-2} \,dx. \end{aligned}$$

Inserting (3.2) into the latter, we derive

3.3 $$\begin{aligned} R^{-2} \int_{B_{2R}} \bigl\vert \nabla(\Delta v) \bigr\vert ^{2}\varphi_{R}^{2m-2} \,dx \leq \epsilon \int_{B_{2R}} \bigl(\Delta^{2} v\bigr)^{2} \varphi_{R}^{2m} \,dx+C_{\epsilon,m} R^{-8} \int_{B_{2R}}v^{2}\varphi_{R}^{2m-8} \,dx. \end{aligned}$$

*Proof of* 3. Integrating by parts, we obtain

$$\begin{aligned} \int_{B_{2R}} \vert \nabla v \vert ^{2} \varphi_{R}^{2m-6} \,dx =&\frac{R^{-6}}{2} \int _{B_{2R}}\Delta\bigl(v^{2}\bigr) \varphi_{R}^{2m-6} \,dx-R^{-6} \int_{B_{2R}}v\Delta v\varphi_{R}^{2m-6} \,dx \\ \leq& \epsilon R^{-4} \int _{B_{2R}}(\Delta v)^{2}\varphi_{R}^{2m-4} \,dx +C_{\epsilon,m} R^{-8} \int_{B_{2R}} v^{2}\varphi_{R}^{2m-8} \,dx. \end{aligned}$$

From (3.2) and (3.3), we deduce

3.4 $$\begin{aligned} R^{-6} \int_{B_{2R}} \vert \nabla v \vert ^{2} \varphi_{R}^{2m-6} \,dx \leq\epsilon^{3} \int _{B_{2R}}\bigl(\Delta^{2} v\bigr)^{2} \varphi_{R}^{2m} \,dx+C_{\epsilon,m} R^{-8} \int _{B_{2R}} v^{2}\varphi_{R}^{2m-8} \,dx. \end{aligned}$$

*Proof of* 4. Integrating by parts, we obtain

3.5 $$\begin{aligned}& R^{-4}\int_{B_{2R}} \bigl\vert \nabla^{2} v \bigr\vert ^{2}\varphi_{R}^{2m-4} \,dx \\& \quad=-R^{-4} \int _{B_{2R}}\nabla v\nabla(\Delta v)\varphi_{R}^{2m-4} \,dx+\frac{R^{-4}}{2} \int _{B_{2R}} \vert \nabla v \vert ^{2}\Delta\bigl( \varphi_{R}^{2m-4}\bigr)\,dx. \end{aligned}$$

Using Young’s inequality and from (3.3) and (3.4), we obtain

$$\begin{aligned} R^{-4} \int_{B_{2R}} \bigl\vert \nabla^{2} v \bigr\vert ^{2}\varphi_{R}^{2m-4} \,dx \leq& R^{-2} \int _{B_{2R}} \bigl\vert \nabla(\Delta v) \bigr\vert ^{2}\varphi_{R}^{2m-2}\,dx +C R^{-6} \int _{B_{2R}} \vert \nabla v \vert ^{2} \varphi_{R}^{2m-6}\,dx \\ \leq& \epsilon^{3} \int_{B_{2R}}\bigl(\Delta^{2} v\bigr)^{2} \varphi _{R}^{2m} +C_{\epsilon,m} R^{-8} \int_{B_{2R}} v^{2}\varphi_{R}^{2m-8}. \end{aligned}$$

*Proof of* 5. Integrating by parts, we get

$$\begin{aligned}& R^{-2} \int_{B_{2R}} \bigl\vert \nabla^{3} v \bigr\vert ^{2}\varphi_{R}^{2m-2} \,dx\\ & \quad =R^{-2} \int _{B_{2R}} \biggl( \bigl\vert \nabla(\Delta v) \bigr\vert ^{2} \varphi_{R}^{2m-2}+ v_{ij} \Delta v_{i} \bigl(\varphi_{R}^{2m-2} \bigr)_{j}+\frac{1}{2} \bigl\vert \nabla^{2} v \bigr\vert ^{2} \Delta\bigl(\varphi _{R}^{2m-2}\bigr) \biggr)\,dx, \end{aligned}$$

where $f_{i}= \frac{\partial f}{\partial x_{i}}$, $f_{ij}= \frac{\partial^{2} f}{\partial x_{j} \partial x_{i}}$ and $f_{ijk}= \frac{\partial^{3} f}{\partial x_{k} \partial x_{j} \partial x_{i}}$. (Here and in the sequel, we use the Einstein summation convention: an index occurring twice in a product is to be summed from 1 up to the space dimension.)

Using Young’s inequality of the above, we deduce

3.6 $$\begin{aligned}& R^{-2} \int_{B_{2R}} \bigl\vert \nabla^{3} v \bigr\vert ^{2}\varphi_{R}^{2m-2} \,dx \\& \quad\leq C R^{-2} \int _{B_{2R}} \bigl\vert \nabla(\Delta v) \bigr\vert ^{2} \varphi_{R}^{2m-2}\,dx+C R^{-4} \int _{B_{2R}} \bigl\vert \nabla^{2} v \bigr\vert ^{2} \varphi_{R}^{2m-4}\,dx, \end{aligned}$$

which gives the desired conclusion. □

### Lemma 3.2

*For any* $m>4$ *and* $\epsilon>0$ *arbitrary small number*, *there exists a constant* $C_{\epsilon,m}>0$ *such that*

3.7 $$\begin{aligned} \bigl(\Delta^{2}\bigl(u\varphi_{R}^{m} \bigr) \bigr)^{2} \leq(1+\epsilon) \bigl(\varphi _{R}^{m} \Delta^{2} u \bigr)^{2}+ C_{\epsilon,m} \mathbf{B}(u, \varphi_{R},m), \end{aligned}$$

*where* $\mathbf{B}(u,\varphi_{R}, m)= ( R^{-4} |\Delta u|^{2}\varphi _{R}^{2m-4} + R^{-2} |\nabla(\Delta u)|^{2}\varphi_{R}^{2m-2} + R^{-6}|\nabla u|^{2}\varphi_{R}^{2m-6} + R^{-8} u^{2} \varphi_{R}^{2m-8} + R^{-4} |\nabla^{2} u|^{2} \varphi_{R}^{2m-4} )$.

### Proof

Let $\varphi_{R} \in C^{4}_{c}(\mathbb {R}^{n})$ be defined as above and $m>4$. Direct calculation yields

3.8 $$ \Delta^{2}\bigl(u\varphi_{R}^{m} \bigr)= \varphi_{R}^{m} \Delta^{2} u+\mathbf{A} \bigl(u, \varphi_{R}^{m}\bigr), $$

where $\mathbf{A}(u, \varphi_{R}^{m})=2\Delta u\Delta\varphi_{R}^{m}+4\nabla u\nabla(\Delta\varphi_{R}^{m})+u\Delta^{2}\varphi_{R}^{m}+4\nabla(\Delta u)\nabla (\varphi_{R}^{m})+4 u_{ij} (\varphi_{R}^{m})_{ij}$.

Thus,

$$\bigl(\Delta^{2}\bigl(u\varphi_{R}^{m}\bigr) \bigr)^{2} = \bigl(\varphi_{R}^{m} \Delta^{2} u \bigr)^{2}+ \mathbf{A}^{2}\bigl(u, \varphi_{R}^{m}\bigr)+ 2 \mathbf{A}\bigl(u, \varphi _{R}^{m}\bigr) \varphi_{R}^{m} \Delta^{2} u. $$

Now by the Young inequality, for any $\epsilon>0$, there exists $C_{\epsilon}$ a constant such that

3.9 $$ \bigl(\Delta^{2}\bigl(u\varphi_{R}^{m} \bigr) \bigr)^{2} \leq(1+\epsilon) \bigl(\varphi _{R}^{m} \Delta^{2} u \bigr)^{2}+ C_{\epsilon} \mathbf{A}^{2}\bigl(u, \varphi_{R}^{m}\bigr). $$

For the second term on the right hand side of inequality (3.9), one obtains

$$\begin{aligned} \mathbf{A}^{2}\bigl(u, \varphi_{R}^{m}\bigr) \leq& C_{\epsilon} \bigl( \vert \Delta u \vert ^{2} \bigl\vert \Delta\varphi_{R}^{m} \bigr\vert ^{2} + \vert \nabla u \vert ^{2} \bigl\vert \nabla\bigl(\Delta \varphi_{R}^{m}\bigr) \bigr\vert ^{2} + \vert u \vert ^{2} \bigl\vert \Delta^{2} \varphi_{R}^{m} \bigr\vert ^{2} \\ &{}+ \bigl\vert \nabla(\Delta u) \bigr\vert ^{2} \bigl\vert \nabla\bigl( \varphi_{R}^{m}\bigr) \bigr\vert ^{2} + \vert u_{ij} \vert ^{2} \bigl\vert \bigl(\varphi_{R}^{m} \bigr)_{ij} \bigr\vert ^{2} \bigr) \\ \leq& C_{\epsilon,m} \bigl( R^{-4} \vert \Delta u \vert ^{2}\varphi_{R}^{2m-4} + R^{-2} \bigl\vert \nabla(\Delta u) \bigr\vert ^{2}\varphi_{R}^{2m-2} + R^{-6} \vert \nabla u \vert ^{2}\varphi _{R}^{2m-6} \\ &{}+ R^{-8} u^{2} \varphi_{R}^{2m-8} + R^{-4} \bigl\vert \nabla^{2} u \bigr\vert ^{2} \varphi _{R}^{2m-4} \bigr), \end{aligned}$$

which gives the desired inequality (3.7). □

Using the previous lemmas, we obtain the following results.

### Proposition 3.1

*Let* $u\in C^{2k}(\mathbb {R}^{n})$ *be a stable solution of* (1.1). *Assume that* *f* *satisfies* $H_{1}$ *and* $H_{2}$. *Then there exists a constant* $C>0$ *such that*, *for any* $R>0$, *we have*

$$\int_{B_{R}} \bigl( \vert u \vert ^{\theta+1}+ \bigl\vert D^{k} u \bigr\vert ^{2} \bigr) \,dx \leq C R^{n- 2k\frac{\theta+ 1}{\theta-1}} \quad\textit{and} \quad \int_{B_{R}} f(u) u \,dx \leq C R^{n- 2k\frac {\theta+ 1}{\theta-1}}. $$

When attempting to prove the nonexistence of the nontrivial solution which is stable outside a compact set of (1.1) in the subcritical case, we need first to establish the following proposition.

### Proposition 3.2

*Let* $u\in C^{2k}(\mathbb {R}^{n})$ *be a solution of* (1.1) *which is stable outside a compact set*. *Assume that* *f* *satisfies* $H_{1}$ *and* $H_{2}$. *Then there exists a constant* $C>0$ *such that*, *for any* $R>0$, *we have*

$$\int_{B_{R}} \bigl( \vert u \vert ^{\theta+1} + \bigl\vert D^{k} u \bigr\vert ^{2} \bigr)\,dx \leq C \bigl(1+ R^{n- 2k\frac{\theta+ 1}{\theta-1}} \bigr) \quad\textit{and} \quad \int_{B_{R}} f(u) u \,dx \leq C \bigl(1+ R^{n- 2k\frac{\theta+ 1}{\theta-1}} \bigr). $$

### Proof of Proposition 3.1

The proof of the case $k=1,2,3$, bears resemblance to an argument found in [5, 6, 8]. For more details, please see the proof of proposition 4 in [6] for the case $k=1$, the proof of Lemma 4.2 in [5] for the case $k=2$ and the proof of Proposition 1.2 in [8] for the case $k=3$. For this reason, we omit the details.

*Proof of the case* $k=4$. Let $\varphi_{ R} \in C^{4}_{c}(\mathbb {R}^{n})$ defined as above, let *u* be a solution of equation (1.1). The function $u \varphi_{ R}^{m} $ belongs to $C^{4}_{c}(\mathbb {R}^{n})$, and thus it can be used as a test function in the quadratic form $Q_{u}$. Hence, the stability assumption on *u* gives

$${ \int_{B_{2R}}}f'(u)u^{2} \varphi_{R}^{2m} \,dx \leq { \int_{B_{2R}}} \bigl\vert \Delta^{2} \bigl(u \varphi_{R}^{m}\bigr) \bigr\vert ^{2} \,dx. $$

Applying Lemma 3.2, we obtain

3.10 $$\begin{aligned} \int_{B_{2R}}f'(u)u^{2} \varphi_{R}^{2m} \,dx \leq&(1+\epsilon) \int_{B_{2R}} \bigl(\varphi_{R}^{m} \Delta^{2} u \bigr)^{2} \,dx+ C_{\epsilon} \int _{B_{2R}}\mathbf{B}(u,\varphi_{R}, m)\,dx. \end{aligned}$$

In view of Lemma 3.1, we get

3.11 $$\begin{aligned} \int_{B_{2R}}f'(u)u^{2} \varphi_{R}^{2m} \,dx \leq& (1+\epsilon) \int _{B_{2R}} \bigl(\varphi_{R}^{m} \Delta^{2} u \bigr)^{2}+ C_{\epsilon}R^{-8} \int_{B_{2R}} u^{2} \varphi_{R}^{2m-8} \,dx. \end{aligned}$$

Multiplying equation (1.1) by $u\varphi_{R}^{2m}$ and integrating by parts, we get

$${ \int_{B_{2R}}}\Delta^{2} u \Delta^{2} \bigl( u \varphi _{R}^{2m} \bigr) \,dx= { \int_{B_{2R}} } f(u)u \varphi_{R}^{2m} \,dx. $$

From (3.8), we derive

$$\begin{aligned}& \int_{B_{2R}} \Delta^{2} u \Delta^{2} \bigl( u \varphi_{R}^{2m} \bigr)\,dx \\& \quad= \int_{B_{2R}} \Delta^{2} u \bigl\{ \bigl( \Delta^{2} u\bigr)\varphi_{R}^{2m}+2\Delta u\Delta \bigl(\varphi_{R}^{2m}\bigr)+4u_{ij} \bigl( \varphi_{R}^{2m}\bigr)_{ij}\\& \qquad{}+4\nabla(\Delta u) \nabla\bigl(\varphi_{R}^{2m}\bigr)+4\nabla u\nabla\bigl( \Delta\bigl(\varphi_{R}^{2m}\bigr)\bigr)+u\Delta ^{2}\bigl(\varphi_{R}^{2m}\bigr) \bigr\} \,dx, \end{aligned}$$

therefore

3.12 $$\begin{aligned} \int_{B_{2R}} \bigl(\bigl(\Delta^{2} u \bigr)^{2}\varphi_{R}^{2m} - f(u)u \varphi _{R}^{2m} \bigr)\,dx =- \int_{B_{2R}}\Delta^{2} u \mathbf{A}\bigl(u, \varphi_{R}^{2m}\bigr)\,dx. \end{aligned}$$

Then, using Young’s inequality, we derive

$$\int_{B_{2R}} \bigl(\bigl(\Delta^{2} u \bigr)^{2}\varphi_{R}^{2m} -f(u)u\varphi _{R}^{2m} \bigr)\,dx \leq \epsilon \int_{B_{2R}} \bigl(\Delta^{2} u\bigr)^{2} \varphi _{R}^{2m} \,dx + C_{\epsilon,m} \int_{B_{2R}}\mathbf{B}(u,\varphi_{R},m)\,dx. $$

Applying again Lemma 3.1, we have

3.13 $$\begin{aligned}& \int_{B_{2R}}\bigl(\Delta^{2} u\bigr)^{2} \varphi_{R}^{2m} \,dx- \int_{B_{2R}} f(u)u\varphi _{R}^{2m} \,dx \\& \quad\leq \epsilon \int_{B_{2R}} \bigl(\Delta^{2} u\bigr)^{2} \varphi_{R}^{2m} \,dx +C_{\epsilon,m} R^{-8} \int_{B_{2R}}u^{2}\varphi_{R}^{2m-8} \,dx. \end{aligned}$$

Multiplying (3.13) by *θ* and combining it with (3.11), we derive

$$\begin{aligned}& \int_{B_{2R}}\bigl[f'(u)u^{2}-\theta f(u)u \bigr]\varphi_{R}^{2m} \,dx+\bigl[\theta(1-\epsilon )-(1+ \epsilon)\bigr] \int_{B_{2R}}\bigl(\Delta^{2} u\bigr)^{2} \varphi_{R}^{2m} \,dx\\& \quad\leq C R^{-8} \int_{B_{2R}}u^{2}\varphi_{R}^{2m-8} \,dx. \end{aligned}$$

From $(H_{1})$ and for *ϵ* sufficiently small such that $\epsilon < \frac{\theta-1}{\theta+1}$, we deduce

3.14 $$\begin{aligned} \int_{B_{2R}}\bigl(\Delta^{2} u\bigr)^{2} \varphi_{R}^{2m} \,dx \leq& C R^{-8} \int _{B_{2R}}u^{2}\varphi_{R}^{2m-8} \,dx. \end{aligned}$$

By Young’s inequality, we have

3.15 $$\begin{aligned} \int_{B_{2R}}\bigl(\Delta^{2} u\bigr)^{2} \varphi_{R}^{2m} \,dx \leq \frac{2}{\theta+1} \int_{B_{2R}}|u|^{\theta+1}\varphi_{R}^{(\theta +1)(m-4)} \,dx+CR^{n-8\frac{\theta+1}{\theta-1}}. \end{aligned}$$

As above, we find from (3.13) that

$$\begin{aligned} \int_{B_{2R}}f(u)u\varphi_{R}^{2m} \,dx \leq& (1+\epsilon) \int_{B_{2R}} \bigl(\Delta^{2} u\bigr)^{2} \varphi_{R}^{2m} \,dx+C_{\epsilon,m} R^{-8} \int _{B_{2R}}u^{2}\varphi_{R}^{2m-8} \,dx. \end{aligned}$$

Using (3.14) in the latter, we obtain

3.16 $$\begin{aligned} \int_{B_{2R}}f(u)u\varphi_{R}^{2m} \,dx \leq& C_{\epsilon}R^{-8} \int _{B_{2R}}u^{2}\varphi_{R}^{2m-8} \,dx \\ \leq& \frac{2}{\theta+1} \int_{B_{2R}}|u|^{\theta+1}\varphi_{R}^{(\theta +1)(m-4)} \,dx+CR^{n-8\frac{\theta+1}{\theta-1}}. \end{aligned}$$

From $(H'_{1})$ and $(H_{2})$, we get

$$\begin{aligned} C_{0} { \int_{B_{2R}}}|u|^{\theta+1}\varphi_{R}^{2m} \,dx \leq& { \int_{B_{2R}}}f(u)u\varphi_{R}^{2m} \,dx \\ \leq& \frac{2}{\theta+1} { \int_{B_{2R}}} \vert u \vert ^{\theta+1}\varphi _{R}^{(\theta+1)(m-4)} \,dx+CR^{n-8\frac{\theta+1}{\theta-1}}, \end{aligned}$$

if $(\theta+1)(m-4)=2m$, then

3.17 $$\begin{aligned} \int_{B_{2R}} \vert u \vert ^{\theta+1} \varphi_{R}^{2m} \,dx\leq CR^{n-8\frac{\theta +1}{\theta-1}}. \end{aligned}$$

From (3.15), (3.16) and (3.17), we deduce that

$${ \int_{B_{2R}}}\bigl(\Delta^{2} u\bigr)^{2} \varphi_{R}^{2m} \,dx\leq CR^{n-8\frac{\theta+1}{\theta-1}},\quad\mbox{and} \quad { \int_{B_{2R}}}f(u)u\varphi_{R}^{2m} \,dx\leq CR^{n-8\frac{\theta+1}{\theta-1}}. $$

Since $\varphi_{R} \equiv1$ in $B_{R}$, we have

$${ \int_{B_{R}}} \bigl(|u|^{\theta+1} + \bigl( \Delta^{2} u\bigr)^{2} \bigr) \,dx\leq CR^{n-8\frac{\theta+1}{\theta-1}},\quad \mbox{and}\quad { \int_{B_{R}}}f(u)u \,dx\leq CR^{n-8\frac {\theta+1}{\theta-1}}. $$

 □

### Proof of Proposition 3.2

The proof of the case $k=1,2,3$, bears resemblance to an argument found in [5, 6, 8]. Now, we prove the case $k=4$. The proof is the same as the proof of Proposition 3.1. We need only to replace $\varphi_{R}$ by $\varphi_{a,R}$, where $\varphi_{a,R}\in C^{4}_{c}(\mathbb {R}^{n})$ satisfies $0\leq\varphi_{a,R} \leq1$ everywhere on $\mathbb {R}^{n}$ such that $\varphi_{a,R}(x)= 0 $ for $|x|< a$ or $|x|>2 R$, $\varphi_{a,R}(x)=1$ for $2a < |x|< R$ and $|\nabla^{\tau} \varphi_{a_{0},R} |\leq C R^{-\tau}$, $\tau \leq4$, for $R < |x|< 2R $. By the stability assumption on *u*, there exists $a_{0}>0$ such that $Q_{u}(u \varphi_{a_{0},R}^{m} )\geq0$ for any $R> 2a_{0}$. Hence, by the choice of the test function $\varphi_{a,R} $, the constant $C_{a_{0}}$ depending on $a_{0}, \epsilon, m$ and *u* appears and the rest of the proof is unchanged. Thus Proposition 3.2 follows. □

As in [20], we shall employ a cut-off function with compact support to derive a variant of the Pohozaev identity. This device allows us to avoid the spherical integrals raised in [21], which are very difficult to control, especially for the polyharmonic situations. For $k=1,2,3$, the Pohozaev identity is similar to [7, 8, 20, 22].

### Proposition 3.3

*Let* $u\in C^{8}(\mathbb {R}^{n})$ *be a solution of* (1.1) *and* $\psi\in C^{4}_{c}(B_{R})$, *then*

3.18 $$\begin{aligned}& \frac{n-8}{2} \int_{B_{R}} \bigl(\Delta^{2} u \bigr)^{2} \psi \,dx - n \int_{B_{R}} F(u) \psi \,dx= \int_{B_{R}} B_{4}(u, \psi) \,dx, \end{aligned}$$

*where*

$$\begin{aligned} B_{4}(u, \psi) =& F(u) \langle x, \nabla\psi \rangle - \frac{1}{2} \bigl(\Delta^{2} u \bigr)^{2} \langle x, \nabla\psi \rangle + 2\Delta^{2} u \nabla \bigl( \bigl\langle x, \nabla( \Delta u) \bigr\rangle \bigr) \nabla\psi \\ & {}+ \Delta^{2} u \bigl\{ \bigl\langle x, \nabla(\Delta u)\bigr\rangle \Delta\psi + 2 \Delta u \Delta\psi \bigr\} + \Delta^{2} u \bigl\{ 4 \nabla(\Delta u) \nabla\psi+ \Delta^{2} \psi \langle x, \nabla u \rangle \bigr\} \\ &{}+ \Delta^{2} u \bigl\{ \Delta\psi \Delta \bigl( \langle x, \nabla u \rangle \bigr) +2 \nabla(\Delta\psi) \nabla \bigl( \langle x, \nabla u \rangle \bigr) + 2 \Delta \bigl[ \nabla \bigl( \langle x, \nabla u \rangle \bigr) \nabla \psi \bigr] \bigr\} . \end{aligned}$$

Thanks to Propositions 3.2 and 3.3, we derive the following.

### Proposition 3.4

*Let* $u\in C^{2k}(\mathbb {R}^{n})$ *be a solution of* (1.1) *which is stable outside a compact set*. *Assume that* *f* *satisfies* $H_{1}$ *and* $H_{2}$. *If* $1 < \theta< p_{s}(n,k)$, *then*

3.19 $$\begin{aligned} \int_{\mathbb {R}^{n}} \bigl| D^{k} u \bigr|^{2} \,dx= \frac{2n}{n-2k} \int_{\mathbb {R}^{n}} F(u) \,dx \end{aligned}$$

*and*

3.20 $$\begin{aligned} \int_{\mathbb {R}^{n}} \bigl\vert D^{k} u \bigr\vert ^{2} \,dx= \int_{\mathbb {R}^{n}} f(u) u \,dx < \infty. \end{aligned}$$

### Proof of Proposition 3.3

Let $u\in C^{8}(\mathbb {R}^{n})$ be a solution of (1.1) and $\psi\in C^{4}_{c}(B_{R})$, we have

$$\Delta \bigl( \langle x, \nabla u \rangle \psi \bigr)= \bigl\langle x,\nabla( \Delta u) \bigr\rangle \psi + 2 \Delta u \psi+ \langle x, \nabla u \rangle \Delta \psi+ 2 \nabla \bigl( \langle x, \nabla u \rangle \bigr) \nabla\psi. $$

Multiplying equation (1.1) by $\langle x, \nabla u \rangle \psi$ and integrating by parts in $B_{R}$, we obtain

3.21 $$\begin{aligned} \int_{B_{R}} f(u) \langle x, \nabla u \rangle \psi \,dx= \int_{B_{R}} \Delta^{3} u \Delta \bigl( \langle x, \nabla u \rangle \psi \bigr) \,dx. \end{aligned}$$

For the right hand side of (3.21), we integrate by parts to get

3.22 $$\begin{aligned}& \int_{B_{R}} \Delta^{3} u \Delta \bigl( \langle x, \nabla u \rangle \psi \bigr) \,dx \\ & \quad= \int_{B_{R}} \Delta^{3} u \bigl( \bigl\langle x,\nabla( \Delta u) \bigr\rangle \psi+ 2 \Delta u \psi + \langle x, \nabla u \rangle \Delta \psi + 2 \nabla \bigl( \langle x, \nabla u \rangle \bigr) \nabla\psi \bigr) \,dx \\ & \quad= \int_{B_{R}} \Delta^{2} u \Delta\bigl[\bigl\langle x, \nabla(\Delta u) \bigr\rangle \bigr] \psi \,dx + 2 \int_{B_{R}} \Delta^{2} u \nabla\bigl[ \bigl\langle x, \nabla(\Delta u) \bigr\rangle \bigr] \nabla\psi \,dx +2 \int _{B_{R}} \bigl(\Delta^{2} u\bigr)^{2} \psi \,dx \\ & \qquad{}+ \int_{B_{R}} \Delta ^{2} u \bigl\{ \bigl\langle x, \nabla(\Delta u) \bigr\rangle \Delta\psi + 2 \Delta u \Delta\psi +4 \nabla(\Delta u) \nabla\psi+ \langle x, \nabla u \rangle \Delta^{2} \psi \bigr\} \,dx \\ & \qquad{}+ \int_{B_{R}} \Delta^{2} u \bigl\{ \Delta\bigl[ \langle x, \nabla u \rangle \bigr]\Delta\psi + 2 \nabla\bigl[ \langle x, \nabla u\rangle \bigr] \nabla(\Delta\psi) \bigr\} \,dx \\ & \qquad {}+ 2 \int _{B_{R}} \Delta^{2} u \Delta\bigl[\nabla \bigl( \langle x, \nabla u\rangle \bigr) \nabla \psi\bigr] \,dx. \end{aligned}$$

For the first term on the right hand side of (3.22), we integrate by parts to find

3.23 $$\begin{aligned}& \int_{B_{R}} \Delta^{2} u \Delta\bigl[ \bigl\langle x, \nabla(\Delta u) \bigr\rangle \bigr] \psi \,dx= \frac{4-n}{2} \int_{B_{R}} \bigl(\Delta^{2} u\bigr)^{2} \psi \,dx- \frac{1}{2} \int _{B_{R}} \bigl(\Delta^{2} u\bigr)^{2} \langle x,\nabla\psi \rangle \,dx. \end{aligned}$$

For the term on the left hand side of (3.22), by integrating by parts, we derive

3.24 $$\begin{aligned} \int_{B_{R}} f(u) \langle x, \nabla u \rangle \psi \,dx =& \int_{B_{R}} \bigl\langle x, \nabla \bigl[F( u)\bigr] \bigr\rangle \psi \,dx \\ =&-n \int_{B_{R}} F( u) \psi \,dx- \int _{B_{R}} F( u) \langle x,\nabla\psi \rangle \,dx . \end{aligned}$$

Therefore, the claim follows from (3.21)-(3.24). □

Here, we are concerned with the proof of Proposition 3.4.

### Proof of Proposition 3.4

*To simplify the proof, we will concentrate on the case* $k = 4$ *which is the most delicate case; even we believe that the results should hold true for* $k=1,2,3$ *, for more details, see for example* [5, 6, 8, 23]. Let $R_{0}>0$. Assume that *u* is stable outside $B_{R_{0}}$. Let $0 < \alpha< \beta$. We begin by defining some smooth compactly supported functions which will be used several times in the sequel. More precisely, we choose $\phi_{R}\in C^{4}_{c}(\mathbb {R}^{n})$ satisfies $0\leq\phi_{R} \leq1$ everywhere on $\mathbb {R}^{n}$ such that

$$\phi_{R}(x)= \textstyle\begin{cases} 1 &\text{for $\alpha R< |x|< \beta R$,}\\ 0&\text{for $|x|< \frac{\alpha}{2}R$ or $|x|> 2 \beta R$,}\\ |\nabla^{k} \phi_{R}|\leq CR^{-k} &\text{on $\{\frac{\alpha}{2}R < |x|< 2 \beta R\}, k=1,2,3,4$}. \end{cases} $$

For *R* large enough such that $\frac{\alpha}{2}R>R_{0}$, then $B_{R_{0}} \cap\{\frac{\alpha}{2}R \leq|x|\leq2 \beta R\}= \varnothing$. Then *u* is stable in $A_{\frac{\alpha}{2}R}^{2 \beta R}:=\{\frac{\alpha }{2}R < |x|< 2 \beta R\}$. By Proposition 3.1, there exists a constant $C>0$ such that

3.25 $$\begin{aligned} \int_{{A^{\beta R}_{\alpha R}}} \bigl( \vert u \vert ^{\theta+1} + \bigl( \Delta^{2} u\bigr)^{2} \bigr) \,dx\leq C R^{n- 8\frac{\theta+ 1}{\theta-1}} \quad \mbox{and}\quad \int_{A^{\beta R}_{\alpha R}} f(u) u \,dx\leq C R^{n- 8\frac{\theta+ 1}{\theta-1}} . \end{aligned}$$

Let $\psi_{R}\in C^{4}_{c}(\mathbb {R}^{n})$ satisfies $0\leq\psi_{R} \leq1$ on $\mathbb {R}^{n}$ defined by

$$\psi_{R}(x)= \textstyle\begin{cases} 1 &\text{for $|x|< \alpha R$,}\\ 0 &\text{for $|x|> \beta R$,}\\ |\nabla^{k} \psi_{R}|\leq CR^{-k} &\text{on $\{\alpha R < |x|< \beta R\}, k=1,2,3,4$}. \end{cases} $$

In view of Lemma 3.1 and Proposition 3.1, we have

3.26 $$\begin{aligned}& \int_{B_{\beta R}} \bigl( \vert u \vert ^{\theta+1} \psi^{2m}_{R} + \bigl(\Delta^{2} u \bigr)^{2} \psi^{2m}_{R} \bigr) \,dx\leq C R^{n- 8\frac{\theta+ 1}{\theta-1}} , \end{aligned}$$

3.27 $$\begin{aligned}& \int_{B_{\beta R}} \bigl( \mathbf{B}(u,\psi_{R}, m) + R^{-2} \bigl|\nabla^{3} u \bigr|^{2} \psi^{2m-2}_{R} \bigr) \,dx\leq C R^{n- 8\frac{\theta+ 1}{\theta-1}}. \end{aligned}$$

Now, we estimate all terms on the right hand side of (3.18). Take $\psi=\psi_{R}^{2m}$ in (3.18), $m>4$.

The second term on the right hand side of (3.18) can be estimated as

3.28 $$\begin{aligned} \biggl\vert - \frac{1}{2} \int_{B_{\beta R}} \bigl(\Delta^{2} u \bigr)^{2} \bigl\langle x, \nabla\psi _{R}^{2m}\bigr\rangle \,dx \biggr\vert =& \biggl\vert - \frac{1}{2} \int_{A^{{\beta R}}_{\alpha R}} \bigl(\Delta^{2} u \bigr)^{2} \bigl\langle x, \nabla\psi_{R}^{2m} \bigr\rangle \,dx \biggr\vert \\ \leq& C_{m} \int_{A^{{\beta R}}_{\alpha R}} \bigl(\Delta^{2} u \bigr)^{2} \psi_{R}^{2m-1} \,dx \leq C R^{ n- 8\frac {\theta+ 1}{\theta-1}}. \end{aligned}$$

Next

3.29 $$\begin{aligned}& \biggl\vert \int_{B_{\beta R}} \bigl[\Delta^{2} u \bigl( \bigl\langle x, \nabla(\Delta u)\bigr\rangle \Delta\psi_{R}^{2m} + 2 \Delta u \Delta\psi_{R}^{2m} +4 \nabla (\Delta u) \nabla \psi_{R}^{2m} + \Delta^{2} \psi_{R}^{2m} \langle x, \nabla u \rangle \bigr) \bigr] \,dx \biggr\vert \\ & \quad= \biggl\vert \int_{A^{{\beta R}}_{\alpha R}} \bigl[ \Delta^{2} u \bigl( \bigl\langle x, \nabla(\Delta u)\bigr\rangle \Delta\psi_{R}^{2m} + 2 \Delta u \Delta\psi_{R}^{2m} +4 \nabla(\Delta u) \nabla \psi_{R}^{2m} + \Delta^{2} \psi_{R}^{2m} \langle x, \nabla u \rangle \bigr) \bigr] \,dx \biggr\vert \\ & \quad\leq C_{m} \int_{A^{\beta R}_{\alpha R}} \bigl\vert \Delta^{2} u \bigr\vert \bigl( R^{-1} \bigl\vert \nabla(\Delta u) \bigr\vert \psi _{R}^{2m-2} + R^{-2} \vert \Delta u \vert \psi_{R}^{2m-2} + R^{-3} \vert \nabla u \vert \psi _{R}^{2m-4} \bigr) \,dx \\ & \quad\leq C_{m} \int_{A^{\beta R}_{\alpha R}} \bigl\vert \Delta^{2} u \bigr\vert \bigl( R^{-1} \bigl\vert \nabla (\Delta u) \bigr\vert \psi_{R}^{m-1} + R^{-2} \vert \Delta u \vert \psi_{R}^{m-2} + R^{-3} \vert \nabla u \vert \psi_{R}^{m-3} \bigr) \,dx, \end{aligned}$$

the last line comes from the fact that $0 \leq\psi_{R} \leq1$, hence $\psi_{R}^{s}\leq\psi_{R}^{t}$, for any $t\leq s$.

By applying the Hölder inequality and the Young inequality to (3.29), we have

3.30 $$\begin{aligned}& \biggl\vert \int_{B_{\beta R}} \bigl[\Delta^{2} u \bigl( \bigl\langle x, \nabla(\Delta u)\bigr\rangle \Delta\psi_{R}^{2m} + 2 \Delta u \Delta\psi_{R}^{2m} +4 \nabla (\Delta u) \nabla \psi_{R}^{2m} + \Delta^{2} \psi_{R}^{2m} \langle x, \nabla u \rangle \bigr) \bigr] \,dx \biggr\vert \\ & \quad\leq \int_{A^{\beta R}_{\alpha R}} \bigl\vert \Delta^{2} u \bigr\vert \bigl( R^{-1} \bigl\vert \nabla(\Delta u) \bigr\vert \psi_{R}^{m-1} + R^{-2} \vert \Delta u \vert \psi_{R}^{m-2} + R^{-3} \vert \nabla u \vert \psi_{R}^{m-3} \bigr) \,dx \\ & \quad\leq \biggl( \int_{A^{\beta R}_{\alpha R}} \bigl(\Delta^{2} u\bigr)^{2} \,dx \biggr)^{\frac{1}{2}} \biggl( \int_{A^{\beta R}_{\alpha R}} \bigl( R^{-1} \bigl\vert \nabla(\Delta u) \bigr\vert \psi_{R}^{m-1} + R^{-2} \vert \Delta u \vert \psi_{R}^{m-2} \\& \qquad {}+ R^{-3} \vert \nabla u \vert \psi_{R}^{m-3} \bigr)^{2} \,dx \biggr)^{\frac{1}{2} } \\ & \quad\leq C \biggl( \int_{A^{\beta R}_{\alpha R}} \bigl(\Delta^{2} u\bigr)^{2} \,dx \biggr)^{\frac{1}{2}} \biggl( \int_{A^{\beta R}_{\alpha R}} \bigl(R^{-2} \bigl\vert \nabla(\Delta u) \bigr\vert ^{2} \psi_{R}^{2m-2} + R^{-4} \vert \Delta u \vert ^{2} \psi _{R}^{2m-4} \\ & \qquad {}+ R^{-6} \vert \nabla u \vert ^{2} \psi_{R}^{2m-6} \bigr) \,dx \biggr)^{\frac{1}{2} }. \end{aligned}$$

Similarly, we also obtain

3.31 $$\begin{aligned}& \biggl\vert \int_{B_{\beta R}} \Delta^{2} u \nabla \bigl(\bigl\langle x, \nabla(\Delta u)\bigr\rangle \bigr) \nabla\psi_{R}^{2m} \,dx \biggr\vert \\& \quad = \biggl\vert \int_{B_{\beta R}} \Delta^{2} u \bigl(\nabla(\Delta u)\nabla \psi_{R}^{2m} + x_{i} (\Delta u)_{ij} \bigl(\psi_{R}^{2m}\bigr)_{j} \bigr) \,dx \biggr\vert \\ & \quad\leq C_{m} \int _{A^{\beta R}_{\alpha R}} \bigl\vert \Delta^{2} u \bigr\vert \bigl(R^{-1} \bigl\vert \nabla(\Delta u) \bigr\vert \psi_{R}^{2m-1} + \bigl\vert (\Delta u)_{ij} \bigr\vert \psi_{R}^{2m-1} \bigr) \,dx \\ & \quad\leq C \biggl( \int_{A^{\beta R}_{\alpha R}} \bigl(\Delta^{2} u\bigr)^{2} \,dx \biggr)^{\frac{1}{2}} \biggl( \int_{A^{\beta R}_{\alpha R}} \bigl(R^{-1} \bigl\vert \nabla(\Delta u) \bigr\vert \psi_{R}^{2m-1} + \bigl\vert (\Delta u)_{ij} \bigr\vert \psi_{R}^{2m-1} \bigr)^{2} \,dx \biggr)^{\frac{1}{2}} \\ & \quad\leq C \biggl( \int_{A^{\beta R}_{\alpha R}} \bigl(\Delta^{2} u\bigr)^{2} \,dx \biggr)^{\frac{1}{2}} \biggl( \int_{A^{\beta R}_{\alpha R}} \bigl( R^{-2} \bigl\vert \nabla(\Delta u) \bigr\vert ^{2} \psi_{R}^{4m-2} + \bigl(( \Delta u)_{ij} \bigr)^{2} \psi_{R}^{4m-2} \bigr) \,dx \biggr)^{\frac{1}{2}}. \end{aligned}$$

Integrating by parts and using Young’s inequality, we obtain

3.32 $$\begin{aligned}& \int_{A^{\beta R}_{\alpha R}} \bigl[ R^{-2} \bigl\vert \nabla(\Delta u) \bigr\vert ^{2} \psi _{R}^{4m-2} + \bigl(( \Delta u)_{ij} \bigr)^{2} \psi_{R}^{4m-2} \bigr] \,dx \\& \quad\leq \int_{B_{\beta R}} \bigl[ R^{-2} \bigl\vert \nabla(\Delta u) \bigr\vert ^{2} \psi _{R}^{4m-2} + \bigl(( \Delta u)_{ij} \bigr)^{2} \psi_{R}^{4m-2} \bigr] \,dx \\& \quad= \int_{B_{\beta R}} \bigl( \Delta^{2} u\bigr)^{2} \psi_{R}^{4m-2} \,dx+ \int_{B_{\beta R}} \Delta^{2} u \nabla(\Delta u ) \nabla \bigl(\psi_{R}^{4m-2}\bigr) \,dx \\& \qquad {}+ \int_{B_{\beta R}} \bigl\vert \nabla(\Delta u ) \bigr\vert ^{2} \biggl[ R^{-2} \psi_{R}^{4m-2} + \frac{1}{2}\Delta \bigl(\psi_{R}^{4m-2}\bigr) \biggr] \,dx \\& \quad \leq C_{m} \int_{B_{\beta R}} \bigl( \Delta^{2} u\bigr)^{2} \psi_{R}^{2m} \,dx + C_{m} R^{-2} \int_{B_{\beta R}} \bigl\vert \nabla(\Delta u ) \bigr\vert ^{2} \psi_{R}^{2m-2}\,dx. \end{aligned}$$

From (3.31) and (3.32), we obtain

3.33 $$\begin{aligned}& \biggl\vert \int_{B_{\beta R}} \Delta^{2} u \nabla \bigl( \bigl\langle x, \nabla(\Delta u) \bigr\rangle \bigr) \nabla\psi_{R}^{2m} \,dx \biggr\vert \\& \quad\leq C \biggl( \int_{A^{\beta R}_{\alpha R}} \bigl(\Delta^{2} u\bigr)^{2} \,dx \biggr)^{\frac{1}{2}} \biggl( \int _{B_{\beta R}} \bigl(\bigl( \Delta^{2} u \bigr)^{2} \psi_{R}^{2m} + R^{-2} \bigl\vert \nabla (\Delta u ) \bigr\vert ^{2} \psi_{R}^{2m-2} \bigr) \,dx \biggr)^{\frac{1}{2}}. \end{aligned}$$

The sixth term on the right hand side of (3.18) yields

3.34 $$\begin{aligned}& \biggl\vert \int_{B_{\beta R}} \Delta^{2} u \bigl( \Delta\bigl( \psi_{R}^{2m}\bigr) \Delta \bigl(\langle x, \nabla u \rangle \bigr) +2 \nabla\bigl(\Delta\bigl(\psi_{R}^{2m}\bigr)\bigr) \nabla \bigl( \langle x, \nabla u \rangle \bigr) \bigr) \,dx \biggr\vert \\& \quad= \biggl\vert \int_{B_{\beta R}} \Delta^{2} u \bigl( \bigl\langle x, \nabla (\Delta u)\bigr\rangle \Delta\bigl(\psi_{R}^{2m}\bigr)+ 2 \Delta u \Delta\bigl(\psi_{R}^{2m}\bigr) \\& \qquad {}+2 \nabla u \nabla \bigl(\Delta\bigl(\psi_{R}^{2m}\bigr)\bigr)+ 2 x_{i} u_{ij} \bigl( \Delta\bigl(\psi_{R}^{2m} \bigr) \bigr)_{j} \bigr) \,dx \biggr\vert \\& \quad\leq C \biggl( \int_{A^{\beta R}_{\alpha R}} \bigl(\Delta^{2} u\bigr)^{2} \,dx \biggr)^{\frac{1}{2}} \biggl( \int_{A^{\beta R}_{\alpha R}} \bigl( R^{-2} \bigl\vert \nabla(\Delta u) \bigr\vert ^{2} \psi_{R}^{2m-2} + R^{-4} (\Delta u )^{2} \psi_{R}^{2m-4} \\& \qquad {}+ R^{-6} \vert \nabla u \vert ^{2} \psi_{R}^{2m-6} + R^{-4} \bigl\vert \nabla^{2} u \bigr\vert ^{2} \psi_{R}^{4m-6} \bigr) \,dx \biggr)^{\frac{1}{2}}. \end{aligned}$$

The last term on the right hand side of (3.18) can be estimated as

$$\begin{aligned}& \int_{B_{\beta R}} \Delta^{2} u \Delta \bigl( \nabla \bigl( \langle x, \nabla u \rangle \bigr) \nabla\bigl(\psi_{R}^{2m} \bigr) \bigr) \,dx \\& \quad= \int_{B_{\beta R}} \Delta ^{2} u \bigl( 3 \nabla(\Delta u) \nabla\bigl(\psi_{R}^{2m}\bigr) + \nabla u \nabla \bigl( \Delta\bigl(\psi_{R}^{2m}\bigr)\bigr) + 2\times \nabla(u_{i}) \times\nabla \bigl(\psi _{R}^{2m} \bigr)_{i} \bigr) \,dx \\& \qquad{}+ \int_{B_{\beta R}} \Delta^{2} u \bigl( x_{i} \times (\Delta u )_{ij} \times \bigl( \psi_{R}^{2m} \bigr)_{j} + u_{ij}\times \bigl\{ x_{i} \bigl( \Delta\bigl(\psi_{R}^{2m}\bigr) \bigr)_{j} + 2 \bigl(\psi_{R}^{2m}\bigr)_{ij} \bigr\} \bigr) \,dx \\& \qquad{}+ 2 \int_{B_{\beta R}} x_{i} \Delta^{2} u \times u_{ijk} \times \bigl(\psi _{R}^{2m} \bigr)_{jk} \,dx. \end{aligned}$$

By Hölder’s inequality and Young’s inequality, we get

3.35 $$\begin{aligned}& \biggl\vert \int_{B_{\beta R}} \Delta^{2} u \Delta \bigl( \nabla \bigl( \langle x, \nabla u\rangle \bigr) \nabla\bigl(\psi_{R}^{2m} \bigr) \bigr) \,dx \biggr\vert \\& \quad\leq C \biggl( \int_{A^{\beta R}_{\alpha R}} \bigl(\Delta^{2} u\bigr)^{2} \,dx \biggr)^{\frac{1}{2}} \\& \qquad{}\times \biggl( \int_{B_{\beta R}} \bigl(R^{-2} \bigl\vert \nabla(\Delta u) \bigr\vert ^{2} \psi_{R}^{4m-2} + R^{-6} \vert \nabla u \vert ^{2} \psi _{R}^{4m-6} +( \Delta u)_{ij}^{2} \times\psi_{R}^{4m-2} \\& \qquad{}+ R^{-4} \bigl\vert \nabla ^{2} u \bigr\vert ^{2} \times\psi_{R}^{4m-6} + R^{-2} \bigl\vert \nabla^{3} u \bigr\vert ^{2} \times\psi_{R}^{4m-4} \bigr) \,dx \biggr)^{\frac{1}{2}} \\& \quad\leq C \biggl( \int_{A^{\beta R}_{\alpha R}} \bigl(\Delta^{2} u\bigr)^{2} \,dx \biggr)^{\frac{1}{2}} \biggl( \int_{B_{\beta R}} \bigl( \bigl(\Delta ^{2} u\bigr)^{2} \psi_{R}^{2m}+ \mathbf{B}(u,\psi_{R}, m) \\& \qquad {}+ R^{-2} \bigl\vert \nabla^{3} u \bigr\vert ^{2} \psi ^{2m-2}_{R} \bigr) \,dx \biggr)^{\frac{1}{2}}. \end{aligned}$$

From hypothesis $H_{1}$, one has $(\theta+1) F(s)\leq f(s)s, \forall s\in \mathbb {R}$. Using the latter inequality, (3.25) and $1<\theta< p_{s}(n,4)$, we get

3.36 $$\begin{aligned} \int_{B_{\beta R}} F(u) \bigl\langle \nabla\psi^{2m}_{R} , x \bigr\rangle \,dx=o(1)\quad \mbox{as } R\rightarrow+ \infty. \end{aligned}$$

From (3.18), (3.25)-(3.36), and $1<\theta< p_{s}(n,4)$, we obtain

$${ \int_{\mathbb {R}^{n}}} \bigl(\Delta^{2} u\bigr)^{2} \,dx=\frac{2n}{n-8} { \int_{\mathbb {R}^{n}}} F(u) \,dx. $$

Now, multiplying equation (1.1) by $u\psi_{R}^{2m}$ and integrating by parts, we get

$$\begin{aligned}& \int_{B_{2R}} \bigl(\bigl(\Delta^{2} u \bigr)^{2}\psi_{R}^{2m} - f(u)u \psi_{R}^{2m} \bigr)\,dx\\& \quad=- \int_{B_{2R}} \Delta^{2} u \bigl(2\Delta u\Delta\bigl( \psi _{R}^{2m}\bigr)+4 u_{ij} \bigl( \psi_{R}^{2m}\bigr)_{ij}+ 4\nabla(\Delta u)\nabla \bigl(\psi _{R}^{2m}\bigr)\\& \qquad{}+4\nabla u\nabla\bigl(\Delta\bigl( \psi_{R}^{2}m\bigr)\bigr)+u\Delta^{2}\bigl( \psi_{R}^{2m}\bigr) \bigr)\,dx. \end{aligned}$$

By the same reasoning as above, we find

$${ \int_{\mathbb {R}^{n}}}\bigl(\Delta^{2} u\bigr)^{2} \,dx = { \int_{\mathbb {R}^{n}}}f(u) u \,dx < \infty. $$

 □

## Proof of Theorems 2.1, 2.2, 2.3, 2.4 and 2.5

### Proof of Theorem 2.1

The proof of Theorem 2.1 for the case $k=1,2,3$ is exactly the same as in [5, 6, 8]. Now, we prove the case $k=4$. Let *u* be a stable solution to (1.1).

*Subcritical case:* $1<\theta<p_{s}(n,4)$ *.* By Proposition 3.1, there exists $C>0$ such that

$$\int_{B_{R}}|u|^{\theta+1} \,dx \leq CR^{n- 8\frac{\theta+1}{\theta-1}}, \quad \forall R>0. $$

Note that

$$n- 8\frac{\theta+1}{\theta-1}=n-8-\frac{16}{\theta-1}< 0, \quad \forall \theta\in \bigl(1, p_{s}(n,4) \bigr). $$

Then, if $1<\theta<p_{s}(n,4)$, after sending $R\rightarrow\infty$, we get $u\equiv0$ in $\mathbb {R}^{n}$.

*Critical case:* $\theta=\frac{n+8}{n-8}$ *.* By Proposition 3.1, we have

$${ \int_{\mathbb {R}^{n}}} \bigl(\bigl(\Delta^{2} u \bigr)^{2} + \vert u \vert ^{\theta+1} \bigr)\,dx < +\infty. $$

So,

4.1 $$\begin{aligned} \lim_{R \rightarrow+ \infty} \int_{A_{R}} \bigl(\bigl(\Delta^{2} u \bigr)^{2} + \vert u \vert ^{\theta+1} \bigr)\,dx=0. \end{aligned}$$

Moreover, if we come back to the proof of Proposition 3.1, we may improve the following integral estimates:

$$\begin{aligned} \int_{B_{R}} \bigl(\bigl(\Delta^{2} u \bigr)^{2} + \vert u \vert ^{\theta+1} \bigr) \,dx \leq C \int_{A_{R}}\bigl(\Delta^{2} u\bigr)^{2} \,dx + C R^{-8} \int_{A_{R}} u^{2} \,dx. \end{aligned}$$

By Hölder’s inequality, we have

$$\begin{aligned} \int_{B_{R}} \bigl(\bigl(\Delta^{2} u \bigr)^{2} + \vert u \vert ^{\theta+1} \bigr) \,dx \leq& C \int_{A_{R}}\bigl(\Delta^{2} u\bigr)^{2} \,dx + C R^{n{\frac{\theta-1}{\theta +1}}-8}\times \biggl( \int_{A_{R}} \vert u \vert ^{\theta+1} \,dx \biggr)^{\frac {2}{\theta+1}} \\ \leq& C \int_{A_{R}}\bigl(\Delta^{2} u\bigr)^{2} \,dx + C \biggl( \int_{A_{R}} \vert u \vert ^{\theta+1} \,dx \biggr)^{\frac{2}{\theta+1}}. \end{aligned}$$

Using (4.1), we get

$${ \int_{\mathbb {R}^{n}}} \vert u \vert ^{\theta+1} \,dx =0. $$

This implies that $u\equiv0$ in $\mathbb {R}^{n}$. □

### Proof of Theorem 2.2

We now collect (3.19) and (3.20). By assumption $H_{3}$, if *u* is not identically zero, then

$$\begin{aligned} \int_{\mathbb {R}^{n}} \bigl\vert D^{k} u \bigr\vert ^{2} \,dx =& \frac{2n}{n-2k} \int_{\mathbb {R}^{n}} F(u)\,dx \geq (1+\alpha_{0}) \int_{\mathbb {R}^{n}}f(u)u \,dx\\ > & \int_{\mathbb {R}^{n}}f(u)u \,dx = \int_{\mathbb {R}^{n}} \bigl\vert D^{k} u \bigr\vert ^{2} \,dx. \end{aligned}$$

This is a contradiction. Then $u\equiv0$. The proof of Theorem 2.2 is thus completed. □

### Proof of Theorem 2.3

The proof of Theorem 2.3 is similar to proof of Proposition 4 in [6]. Let $\gamma\in [1, 2\theta-1+2\sqrt{\theta(\theta-1)})$. Multiply equation (1.1) by $|u|^{\gamma-1}u \varphi_{R}^{2}$ and integrate by parts to find

4.2 $$\begin{aligned}& \int_{B_{2R}} f(u) u \vert u \vert ^{\gamma-1} \varphi_{R}^{2} \,dx \\ & \quad= \frac{4 \gamma }{(\gamma+1)^{2}} \int_{B_{2R}} \bigl|\nabla\bigl( \vert u \vert ^{\frac{\gamma -1}{2}}u \bigr)\bigr|^{2}\varphi_{R}^{2} \,dx -\frac{1}{\gamma+1} \int_{B_{2R}} \vert u \vert ^{\gamma +1} \Delta \bigl( \varphi_{R}^{2} \bigr) \,dx. \end{aligned}$$

The function $|u|^{\frac{\gamma-1}{2}}u \varphi_{R} \in C^{1}_{c}(\mathbb {R}^{n})$, and thus it can be used as a test function in the quadratic form $Q_{u}$. Hence, the stability assumption on *u* gives

$$\begin{aligned}& \int_{B_{2R}} f'(u) \vert u \vert ^{\gamma+1} \varphi_{R}^{2} \,dx \\& \quad\leq \int_{B_{2R}} \bigl\vert \nabla\bigl( \vert u \vert ^{\frac{\gamma-1}{2}}u\bigr) \bigr\vert ^{2}\varphi_{R}^{2} \,dx + \int_{B_{2R}} \vert u \vert ^{\gamma+1} \vert \nabla \varphi_{R} \vert ^{2} \,dx - \frac{1}{2} \int_{B_{2R}} \vert u \vert ^{\gamma+1} \Delta \bigl( \varphi_{R}^{2} \bigr) \,dx. \end{aligned}$$

Using (4.2) in the latter, we obtain

$$\begin{aligned}& \int_{B_{2R}} \biggl\{ \bigl(f'(u)u^{2} - \theta f(u) u \bigr) |u|^{\gamma -1} + \biggl( \frac{4 \gamma\theta}{(\gamma+1)^{2}} -1 \biggr)\bigl| \nabla \bigl(|u|^{\frac{\gamma-1}{2}}u\bigr)\bigr|^{2} \biggr\} \varphi_{R}^{2} \,dx \\& \quad\leq C_{1}(\gamma , \theta) \int_{B_{2R}} |u|^{\gamma+1} \Delta \bigl( \varphi_{R}^{2} \bigr) \,dx + \int_{B_{2R}} |u|^{\gamma+1} |\nabla\varphi_{R}|^{2} \,dx, \end{aligned}$$

where $C_{1}(\gamma, \theta)= (\frac{\theta}{\gamma+1} - \frac{1}{2} )$. By hypothesis $H_{1}$, we obtain

$$\begin{aligned}& \biggl( \frac{4 \gamma\theta}{(\gamma+1)^{2}} -1 \biggr) \int_{B_{2R}} \bigl\vert \nabla\bigl( \vert u \vert ^{\frac{\gamma-1}{2}}u\bigr) \bigr\vert ^{2}\varphi_{R}^{2} \,dx \\& \quad\leq C_{1}(\gamma, \theta) \int_{B_{2R}} \vert u \vert ^{\gamma+1} \Delta \bigl( \varphi_{R}^{2} \bigr) \,dx + \int_{B_{2R}} \vert u \vert ^{\gamma+1} \vert \nabla \varphi_{R} \vert ^{2} \,dx. \end{aligned}$$

Since $\theta>1$ and $\gamma\in[ 1, 2\theta-1+2\sqrt{\theta(\theta -1)})$, we have $\frac{4 \gamma\theta}{(\gamma+1)^{2}} -1>0$ and

$$\begin{aligned} \int_{B_{2R}} \bigl\vert \nabla\bigl( \vert u \vert ^{\frac{\gamma-1}{2}}u \bigr) \bigr\vert ^{2}\varphi_{R}^{2} \,dx \leq C(\gamma, \theta) \int_{B_{2R}} \vert u \vert ^{\gamma+1} \bigl( \bigl\vert \Delta \bigl(\varphi_{R}^{2} \bigr) \bigr\vert + \vert \nabla\varphi_{R} \vert ^{2} \bigr)\,dx. \end{aligned}$$

Using again (4.2), we get

$${ \int_{B_{2R}}} f(u) u \vert u \vert ^{\gamma-1} \varphi_{R}^{2} \,dx \leq C'(\gamma, \theta) { \int_{B_{2R}}} \vert u \vert ^{\gamma+1} \bigl( \vert \nabla\varphi_{R} \vert ^{2} + \bigl\vert \Delta \bigl( \varphi_{R}^{2} \bigr) \bigr\vert \bigr)\,dx. $$

First, we replace $\varphi_{R}$ by $\varphi_{R}^{m}$ in the latter inequality, for any $m>2$, we derive

$$\begin{aligned} \int_{B_{2R}} f(u) u \vert u \vert ^{\gamma-1} \varphi_{R}^{2m} \,dx \leq& C(\gamma, \theta,m) \int_{B_{2R}} \vert u \vert ^{\gamma+1} \varphi_{R}^{2m-2} \bigl( \vert \nabla \varphi_{R} \vert ^{2} + \vert \Delta\varphi_{R} \vert \bigr) \,dx \\ \leq& \frac {C}{R^{2}} \int_{B_{2R}} \vert u \vert ^{\gamma+1} \varphi_{R}^{2m-2}\,dx. \end{aligned}$$

By $H_{1}$ and $H_{2}$, we get

$${ \int_{B_{2R}}} \vert u \vert ^{\theta+\gamma} \varphi_{R}^{2m} \,dx \leq \frac{C}{R^{2}} \int_{B_{2R}} \vert u \vert ^{\gamma+1} \varphi_{R}^{2m-2} \,dx. $$

An application of Young’s inequality yields

$${ \int_{B_{2R}}} \vert u \vert ^{\theta+\gamma} \varphi_{R}^{2m} \,dx \leq C R^{n- 2\frac{\theta+ \gamma}{\theta-1}}+ \frac{\gamma+1}{\gamma+\theta } { \int_{B_{2R}}} \vert u \vert ^{\gamma+\theta} \varphi _{R}^{(2m-2)\frac{\gamma+\theta}{\gamma+1}}\,dx. $$

Thus

$${ \int_{B_{R}}} \vert u \vert ^{\theta+\gamma} \,dx \leq C' R^{n- 2\frac {\theta+ \gamma}{\theta-1}}. $$

As in Farina’s work we readily deduce, by letting $R\to+\infty$, that there is no nontrivial stable solution of (1.1), in the special case $1< \theta<p_{c}(n)$. □

### Proof of Theorem 2.4

We proceed as in the proof of Proposition 2.1. From (3.11) and (3.13), we deduce by replacing $f(u)$ by $-m u+ \lambda |u|^{\theta-1}u-\mu|u|^{p-1}u$ that

4.3 $$\begin{aligned}& (1-\epsilon) \int_{B_{2R}}\bigl(\Delta^{2} u\bigr)^{2} \varphi_{R}^{2m} \,dx- \int _{B_{2R}}\bigl(-m u^{2} + \lambda \vert u \vert ^{\theta+1}-\mu \vert u \vert ^{p+1}\bigr)\varphi_{R}^{2m} \,dx \\& \quad\leq C_{\epsilon}R^{-8} \int_{B_{2R}}u^{2}\varphi_{R}^{2m-8} \,dx \end{aligned}$$

and

4.4 $$\begin{aligned}& \int_{B_{2R}}\bigl(-m u^{2} + \theta\lambda \vert u \vert ^{\theta+1}-p \mu \vert u \vert ^{p+1}\bigr) \varphi_{R}^{2m} \,dx \\& \quad\leq (1+\epsilon) \int_{B_{2R}} \bigl(\varphi_{R}^{m} \Delta^{2} u \bigr)^{2}+ C_{\epsilon}R^{-8} \int_{B_{2R}} u^{2} \varphi_{R}^{2m-8} \,dx. \end{aligned}$$

Multiplying (4.3) by *θ* and combining it with (4.4), we derive

$$\begin{aligned}& m(\theta-1) \int_{B_{2R}} u^{2} \varphi_{R}^{2m} \,dx + \mu(\theta-p) \int _{B_{2R}} \vert u \vert ^{p+1} \varphi_{R}^{2m} \,dx\\& \qquad{}+\bigl[\theta(1-\epsilon)-(1+\epsilon ) \bigr] \int_{B_{2R}}\bigl(\Delta^{2} u\bigr)^{2} \varphi_{R}^{2m} \,dx\\& \quad\leq C R^{-8} \int _{B_{2R}}u^{2}\varphi_{R}^{2m-8} \,dx. \end{aligned}$$

For *ϵ* sufficiently small, we deduce

4.5 $$\begin{aligned}& m(\theta-1) \int_{B_{2R}} u^{2} \varphi_{R}^{2m} \,dx + \mu(\theta-p) \int _{B_{2R}} \vert u \vert ^{p+1} \varphi_{R}^{2m}\,dx + \int_{B_{2R}}\bigl(\Delta^{2} u\bigr)^{2} \varphi _{R}^{2m} \,dx \\& \quad\leq C R^{-8} \int_{B_{2R}}u^{2}\varphi_{R}^{2m-8} \,dx. \end{aligned}$$

*Proof of* 1. If $m>0$ and $\theta\geq p$, then from (4.5), we deduce that

$$\begin{aligned} \int_{B_{R}} u^{2} \,dx \leq C R^{-8} \int_{B_{2R}}u^{2} \,dx. \end{aligned}$$

Let $J(R):= {\int_{B_{R}}} u^{2} \,dx $. If we iterate the above inequality, then we get

4.6 $$\begin{aligned} J(R)\leq C R^{-8(k+1)} J\bigl(2^{k+1} R\bigr). \end{aligned}$$

We deduce from the boundedness of *u* that the right hand side of (4.6) is of order $R^{M}$ with $M= -8 (k+1)+n \rightarrow0$ as $k\rightarrow+ \infty$. Hence, we can choose *k* large enough such that $M<0$. Then it follows from (4.6) that $J(R)\rightarrow 0, \mbox{as} R \rightarrow+\infty$. So we get

$${ \int_{\mathbb {R}^{n}}} u^{2} \,dx=0. $$

Then $u \equiv0$.

*Proof of* 2. If $\theta> p$, $m\geq0$, then from (4.5) and by Young’s inequality, we get

$$\begin{aligned} \int_{B_{2R}} \vert u \vert ^{p+1} \varphi_{R}^{2m} \,dx + \int_{B_{2R}}\bigl(\Delta^{2} u\bigr)^{2} \varphi_{R}^{2m} \,dx \leq\frac{2}{p+1} \int_{B_{2R}} \vert u \vert ^{p+1}\varphi _{R}^{(p+1)(m-4)} \,dx+CR^{n-8\frac{p+1}{p-1}}. \end{aligned}$$

Choosing $2m=(p+1)(m-4)$, thus

$${ \int_{B_{2R}}} \vert u \vert ^{p+1} \varphi_{R}^{2m} \,dx+ { \int_{B_{2R}}}\bigl(\Delta^{2} u\bigr)^{2} \varphi_{R}^{2m} \,dx\leq CR^{n-8\frac{p+1}{p-1}}. $$

Consequently

$${ \int_{B_{R}}} \vert u \vert ^{p+1} \,dx+ { \int _{B_{R}}}\bigl(\Delta^{2} u\bigr)^{2} \,dx \leq C R^{n-8\frac{p+1}{p-1}} . $$

The result then follows in a similar way to that in the proof of Theorem 2.1. This completes the proof of Theorem 2.4. □

### Proof of Theorem 2.5

We can proceed as in the proof of Proposition 3.4, we get

$${ \int_{\mathbb {R}^{n}}} \bigl\vert D^{k} u \bigr\vert ^{2}=\frac{2n}{n-2k} { \int_{\mathbb {R}^{n}}} \biggl( - \frac{m}{2} u^{2} + \frac{\lambda}{\theta+1} \vert u \vert ^{\theta+1} - \frac{\mu}{p+1} \vert u \vert ^{p+1} \biggr) $$

and

$${ \int_{\mathbb {R}^{n}}} \bigl\vert D^{k} u \bigr\vert ^{2}= { \int_{\mathbb {R}^{n}}} \bigl( - m u^{2} + \lambda \vert u \vert ^{\theta+1} - \mu \vert u \vert ^{p+1} \bigr). $$

Thus

$$\begin{aligned}& \frac{2mk}{n-2k} { \int_{\mathbb {R}^{n}}} u^{2} \,dx + \lambda \biggl( 1- \frac{2n}{(n-2k)(\theta+1) } \biggr) { \int_{\mathbb {R}^{n}}} \vert u \vert ^{\theta+1} \,dx \\& \quad{}+\mu \biggl( \frac{2n}{(n-2k)(p +1) } -1 \biggr) { \int_{\mathbb {R}^{n}}} \vert u \vert ^{p+1} \,dx = 0. \end{aligned}$$

This concludes the proof of Theorem 2.5. □
